# Using Network Analysis to Understand Knowledge Mobilization in a Community-based Organization

**DOI:** 10.1007/s12529-014-9430-6

**Published:** 2014-09-04

**Authors:** Heather L. Gainforth, Amy E. Latimer-Cheung, Spencer Moore, Peter Athanasopoulos, Kathleen A. Martin Ginis

**Affiliations:** 1 University College London, 1-19 Torrington Place, London, WC1E 7HB UK; 2 Queen’s University, 28 Division Street, Kingston, Ontario K7L 3N6 Canada; 3 Spinal Cord Injury Ontario, 520 Sutherland Drive, Toronto, Ontario M4G 3V9 Canada; 4 McMaster University, 1280 Main St. West, Hamilton, Ontario L*S4L8 Canada

**Keywords:** Network analysis, Community-based Organization, Knowledge mobilization, Knowledge translation, Physical activity

## Abstract

**Background:**

Knowledge mobilization (KM) has been described as putting research in the hands of research users. Network analysis is an empirical approach that has potential for examining the complex process of knowledge mobilization within community-based organizations (CBOs). Yet, conducting a network analysis in a CBO presents challenges.

**Purpose:**

The purpose of this paper is to demonstrate the value and feasibility of using network analysis as a method for understanding knowledge mobilization within a CBO by (1) presenting challenges and solutions to conducting a network analysis in a CBO, (2) examining the feasibility of our methodology, and (3) demonstrating the utility of this methodology through an example of a network analysis conducted in a CBO engaging in knowledge mobilization activities.

**Method:**

The final method used by the partnership team to conduct our network analysis of a CBO is described.

**Results:**

An example of network analysis results of a CBO engaging in knowledge mobilization is presented. In total, 81 participants completed the network survey. All of the feasibility benchmarks set by the CBO were met. Results of the network analysis are highlighted and discussed as a means of identifying (1) prominent and influential individuals in the knowledge mobilization process and (2) areas for improvement in future knowledge mobilization initiatives.

**Conclusion:**

Findings demonstrate that network analysis can be feasibly used to provide a rich description of a CBO engaging in knowledge mobilization activities.

## Introduction

Knowledge mobilization (KM) has been described as putting research in the hands of research users [1]. Engaging in KM ensures that the resources and time that have been devoted to conducting research are not wasted and that beneficial interventions, treatments and policies are accessible to the general population [2, 3]. A unique approach that has promise for ensuring that the benefits of KM reach the public is using community-based organizations (CBOs) to help disseminate and implement evidence-based public health programs [4–6]. CBOs are not-for-profit organizations that have a mandate to provide programs and services to individuals in their community who are often marginalized and/or stigmatized (e.g. persons with disabilities and/or people living with HIV/AIDS) [6]. Thus, a CBO has potential to be an excellent conduit of information to the individuals they serve. Despite CBOs’ strategic role in the public health system, few studies have examined how KM occurs within CBOs [6]. To encourage KM in CBOs, further efforts are needed to understand the process of KM within CBOs.

Network analysis is an empirical approach that can be used to examine the complex process of KM within CBOs. Network analysis provides a valuable set of theories, tools and methods for describing, exploring and understanding the structural and relational aspects of a group (i.e. a CBO) [7]. In particular, network analysis can provide an understanding of how knowledge flows (or fails to flow) within an organization. This information can be used to understand the process of KM within the organization, design future KM initiatives and provide members of the CBO with valuable information about how their network is functioning and evolving [8, 9].

There is an emerging literature of network analysis research in the field of KM. Knowledge mobilization network analyses examining the adoption of evidence-based practice have been conducted within a coalition of community groups, a public health department, health promotion programs and youth services [8, 10–13]. While this research begins to describe KM in organizations, a network analysis within a CBO presents several unique methodological and ethical challenges that have not been addressed in previous research. To complete a network questionnaire, participants need to name individuals or organizations in their network. CBOs provide confidential services that assist clients in their daily lives. Disclosing this confidential information (i.e. names and affiliations) can break existing confidentiality agreements between the CBO and its clients as well as expose an individual’s job performance to managerial scrutiny [10]. Furthermore, network analysis is relatively unfamiliar to the public [10]. While members of the CBO may have considerable experience filling out survey questionnaires, it is unlikely that they have experience completing a network questionnaire. Therefore, participants may be unaware of the potential consequences of disclosing confidential information in the network questionnaire. This inexperience completing network questionnaires may also lead to participant burden and the potential for nonresponse bias. Being able to understand, navigate and address these challenges is an essential first step for ensuring the viability of using network analysis to understand KM within CBOs [14].

The aim of this paper is to demonstrate the value and feasibility of using network analysis as a method for understanding KM within a CBO. To achieve this aim, we present a network analysis of a CBO undergoing KM activities to disseminate the physical activity guidelines to people with spinal cord injury (SCI). This paper is divided into four sections. First, we present the challenges that emerged when conducting the network analysis within the CBO and the solutions used to address these challenges. Second, we present a method for conducting network analysis that addresses ethical and practical concerns of a CBO. Third, we examine the feasibility of conducting network analysis within a CBO using our approach. Finally, we present an example of the results of our network analysis as a demonstration of the utility of network analysis for the CBO and researchers.

## Context

Spinal cord injury (SCI) results from damage to the spinal cord due to trauma or disease and leads to partial or complete paralysis. While adopting a physical active lifestyle offers several benefits to people with SCI, physical activity promotion initiatives for the SCI population are sparse, and physical activity participation rates among people with SCI are low [15, 16]. In 2011, the first evidence-based physical activity guidelines for people with SCI were released [17]. These guidelines are novel in that they are the first systematically developed, evidence-based guidelines to outline the amount, intensity and types of activity required to obtain fitness benefits for a special population (see http://www.sciactioncanada.ca/guidelines/) [17]. To optimize dissemination of the guidelines, the guideline developers (SCI Action Canada) forged a partnership with a CBO (SCI Ontario) that assists people with SCI and other physical disabilities. SCI Ontario consists of 17 regional offices across the province of Ontario, 8 different client programs and services, 13 departments in the core areas of Peer Support, Regional Services, Membership, Employment Services, Advocacy, Information Services and Attendant Services [18]. Together, these two partner organizations undertook multiple initiatives to disseminate the guidelines widely [4]. These initiatives aimed to inform all SCI Ontario staff (i.e. CBO staff) and volunteers as well as people with SCI about the guidelines and are described elsewhere [4, 19–21]. As part of this comprehensive effort, there was the opportunity to use network analysis methods to examine the dissemination of a novel innovation, i.e. the SCI physical activity guidelines, within a CBO. In particular, all of the relations between staff (*n* = 71) and volunteers (*n* = 278) who worked within the service provision branch of the organization were invited to participate. The guidelines were relevant to these individuals because both staff and volunteers work to assist clients of the CBO who have an SCI or a physical disability in the transition from acute care through rehabilitation and back into the community. Therefore, all staff and volunteers were encouraged to share information about the guidelines.

## Part 1: Development of the Network Instrument and Procedures

The researchers and the CBO administrators worked in a community-university collaborated over the course of 6 months to develop the network analysis methodology. Initial meetings between the researchers and the CBO administration focused on determining the appropriateness of a network analysis approach for understanding the mobilization of the new physical activity guidelines within the organization. Critical to the project’s success was the researchers and CBO’s recognition of the mutual benefits of such an analysis. Support for the network analysis was gained through discussions with and presentations to all of the management staff about the study’s potential benefits to the organizations. CBO management staff saw the analysis as beneficial because it would (1) provide insight into the organization’s current communication methods, (2) provide recommendations for improving communication within the CBO, and (3) establish a template for future KM efforts.

After gaining support for the analysis, the dialogue shifted to focus on the ethical and methodological challenges of conducting a network analysis within the context of the organization. The primary concerns of the partnership team were ensuring that conducting a network analysis would not (1) breach the confidentiality of the CBO clients, (2) violate ethics policies set out by the researchers’ institutional ethics review boards and the CBO, and (3) be taxing for the staff and volunteers at the CBO. The solutions established by the partnership are described below.

### Solution to Ethical and Methodological Considerations

#### Concern 1: Maintaining the Confidentiality of the CBO’s Clients

Collecting network data within the CBO required participants to provide their name and the names and affiliations of individuals (e.g. clients, staff, volunteers) or organizations within their network. Such information enables examination of the ties among the various members of the network. Disclosure of potentially confidential information may dissuade some CBOs or individuals in the network from participating or completing the network questionnaire honestly [10]. Lack of participation and dishonest responses are particularly problematic within network analysis because results are often dependent on knowing the links among all members of a network. Nonparticipation and response bias can become challenging in CBOs that have previously established confidentiality agreements with clients because providing client names would breach these agreements.

To ensure that the organization’s confidentiality agreements were not breached, the partnership team decided to ask participants not to provide their clients’ real names and to indicate only the frequency of interactions with their clients. By adopting this approach, we were able to ensure that client names remained confidential while still collecting the required information.

#### Concern 2: Ensure that the CBO Understands the Ethical Standards of the Data Analysis

The ethical standards to which researchers adhere can be quite unfamiliar to CBOs. Particularly relevant to network analysis, the CBO was concerned about how researchers would address the ethical issues of (1) anonymity because names or pseudonyms must be recorded for the network data to be meaningful and (2) nonparticipation because even if a respondent does not consent to complete the network questionnaire, information about them might still be collected through participant responses to the network questions [10].

To help the CBO understand the ethical standards to which researchers would adhere to in the present study, documentation was provided to the CBO staff detailing (1) how network data would be analyzed and handled by the researchers and the CBO and (2) the ethical standards of the researchers’ institutional ethics review board. Of particular concern to the CBO and the researchers was that results of the network analysis would not identify individual staff and volunteers. The issue of anonymity was addressed by ensuring that the names of participants were stripped from the data file and replaced with ID codes once all data had been entered into the network software. Furthermore, it was decided that only research members of the partnership team would be able to see the nonanonymous data. The issue of nonparticipation was dealt with by clarifying to the CBO that participants are reporting on their *perception* of a relationship with another person. Therefore, respondents are not being unethical by reporting the names of individuals in their network as they have a right to report their own perceptions.

#### Concern 3: Minimize Participant Burden

Given that network analysis is less common than other questionnaire-based methodologies, participants may be unaware of the ethical consequences of completing a network questionnaire, may not know how to complete the questionnaire, and may find the questionnaire burdensome to complete. To overcome this challenge, the partnership team ensured that (1) extensive training was offered to participants regarding how to complete a network questionnaire and the implications of participating and (2) that participants could easily access and complete the network questionnaire.

The partnership team ensured that sufficient education and training about network analysis were offered to all potential participants. First, the partnership team chose a representative to work directly with the CBO’s chief executive officer (CEO) to create educational e-mails that were sent to all potential participants by the CEO. Second, the CBO representative held meetings with its administration and department managers. Within these meetings, managers were provided with information about the implications of the network analysis for the organization and the study procedure. Managers were encouraged to liaise with their staff about the network analysis. Finally, the partnership team developed a step-by-step instructional video that demonstrated how to complete the network survey online using a hypothetical example. This instructional video was embedded into the online survey.

Our partnership team also ensured that the network questionnaires were simple and not burdensome for participants to complete. First, the partnership team decided that the network survey should be conducted online to allow participants to complete the survey at their convenience. Second, the partnership team decided to create two versions of the online survey. One survey was created for staff and another for volunteers. While the network surveys were almost identical, the terminology specific to participants’ role as a staff member or a volunteer with the organization was used. Also, the step-by-step instructional video was tailored to participants’ role as a staff member or volunteer. Finally, prior to the release of the network survey to the organization, the face validity of the online network survey was assessed by members of the CBO’s administrative staff. They reviewed the surveys for ease of use and understanding. All administrator reviewers deemed both versions of the network survey to be acceptable.

## Part 2: Methodology Used to Conduct the Network Analysis

Below, the final method used by the partnership team to conduct our network analysis of a CBO is described, and an example of our results is presented.

### Design and Sample

A cross-sectional design and whole-network design (i.e. collected information about each individual’s ties with all other actors) were used to investigate all of the relations between CBO staff member (*n* = 71) and volunteers (*n* = 278) who work within the CBO’s service provision branch. These individuals work to assist clients who have an SCI or a physical disability in the transition from acute care through rehabilitation and back into the community. In the current analysis, the focal point was the relationship between individuals exchanging information or sharing resources to advance physical activity knowledge and participation among Canadians living with SCI.

### Questionnaires

#### Demographic Information

All participants indicated their age, gender, education and the number of years they worked (volunteered) in their current position. Participants who were staff members also indicated the department in which they worked. If participants indicated having an SCI, they were asked to indicate their number of years post injury, level and cause of SCI. Participants were also asked to indicate their name.

#### Network Instrument

At the time the network analysis was conducted, KM activities had been occurring within the CBO for 7 months. Therefore, participants were asked about sharing information about physical activity for people with SCI in the last 7 months. Sharing information about physical activity for people with SCI was specifically defined as receiving information and/or providing information about physical activity for people with SCI. To avoid recall bias, the organization asked staff and volunteers to keep written and digital records of information sharing. To maintain clarity, the network instrument was divided into four sections: (1) clients, (2) people within the CBO, (3) people outside of the CBO and (4) resources. Sections 2 and 3 were used to identify actors in the network.

The first section on sharing information about physical activity with clients had three questions. To maintain client anonymity, clients were not surveyed, and participants were specifically told to indicate only frequencies and not the names of clients. Specifically, participants were asked to indicate the number of clients that (1) they had spoken to about physical activity, (2) had asked them about physical activity and (3) they had worked with. These measures were not included in the network but used an outcome variable examining adoption of behaviours to promote the guidelines. In the second and third section, participants were allowed to use names and were asked to freely recall the names of people within and outside of the CBO with whom they had shared information about physical activity in the last 7 months. Participants were free to name as many people as they wished. For each name generated, participants were asked to indicate (1) the role the person played and (2) how they shared information with the person (received information, provided information, both received and provided information). The final section included four questions which assessed the resources that participants used to gain information about physical activity. The first three questions asked participants whether they had (1) read the articles about physical activity for people with SCI in the CBO’s magazine, (2) used the CBO’s website to access information about physical activity, (3) accessed the SCI Action Canada website. The fourth question asked participants to list any other resources they had used to access information about physical activity for people with SCI.

## Part 3: The Feasibility of Conducting a Network Analysis in a CBO

Feasibility of the network analysis was examined using benchmarks set by the CBO for participation rates, representativeness and administrative demands associated with the network analysis. Regarding participation rates, the CBO felt that it would be acceptable if 80 % of staff participated in the survey. This aligns with network research indicating that response rate of 75 % or higher limits the potential negative effects of missing data in a whole-network approach [22]. While volunteers provided important insight, the CBO staff felt that setting a benchmark for volunteers was not appropriate given that their primary target for recruitment and education was staff. Therefore, the CBO deemed any participation by volunteers in the network survey as successful. In terms of representativeness, the CBO set the benchmark that the majority of staff who completed the survey needed to work primarily with clients. Notably, service provision staff that did not work directly with clients were also included in the analysis as these individuals needed to be able to communicate with front line staff and volunteers about the guidelines and refer to guidelines in publications and reports (e.g. the magazine produced by the CBO). A benchmark for representativeness for volunteers was not set as all volunteers work directly with clients. Finally, the benchmark set by the CBO for administrative demands was the minimization of participant and administrative burden. Measures of participant and administrative demand included the number of participants who contacted the researchers for assistance to complete the network survey, the time needed to develop the network instrument, the time needed to develop the instructional video and the recruitment period. In terms of participation rates, 79 % of staff and 9 % of volunteers completed the network survey. Regarding representativeness, the benchmark set by the CBO was met as the majority of staff worked directly with clients (61 %).

In terms of the data collection process, the majority of participants were able to complete the network questionnaire and very few complications arose. Only 4 % of participants contacted the researchers or service organization administrators to indicate that they needed assistance completing the network questionnaire. The occurrence of names not matching with 100 % accuracy was not frequent. All discrepancies were related to the spelling of actors’ names and were easily resolved by checking the name provided against the CBOs’ roster. In total, it took 6 months to design a network instrument that could address the methodological and ethical concerns of the partnership team. Once the survey was developed, 1 month was needed to create the instructional video, and the recruitment period lasted approximately 1 month.

## Part 4: Example Results of the Network Analysis

### Data Analysis

**Fig. 1:**
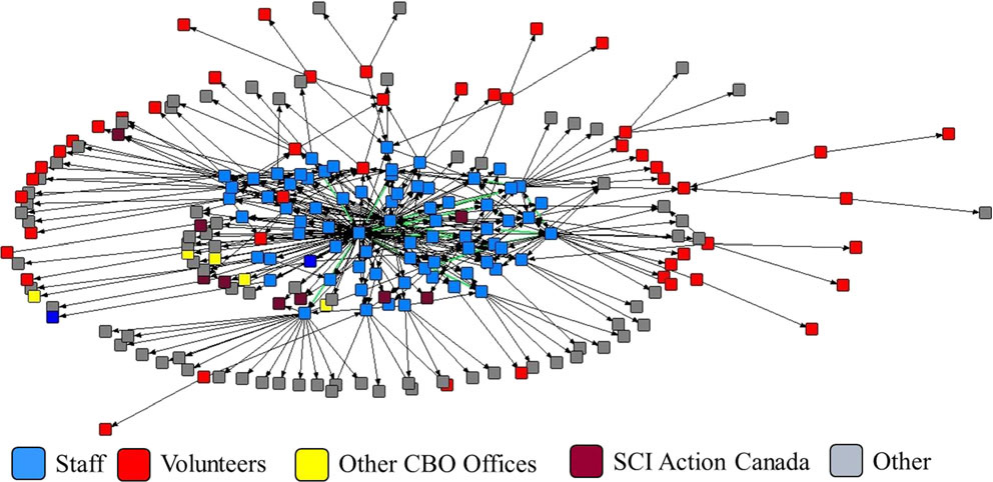
Sociogram depicting the network structure of the CBO. Green lines denote reciprocal ties within the network

The purpose of the network analysis was to determine the network structure of a CBO engaging in KM. Because we were interested in the role of volunteers in the organization, volunteers were included in the analysis despite their low response rate. In subsequent analyses examining how the network structure was associated with the adoption of evidence-based practice, a sub-analysis of only staff was used. The results parallel the network structure presented in this paper and are described elsewhere [23]. The network analysis was performed using UCINET v6 [24] and NETDRAW [25] software. The resulting sociogram is presented in Fig. 1. Because we were interested in how information was being shared, we examined our network data as a directed, asymmetric network. Network and individual level measures were calculated to describe individual and network attributes. Given the unique context of conducting a network analysis examining KM in a CBO that serves a special population, formal hypotheses relating to the network structure were not put forth. Table 1 defines each of the measures and how these measures were used by the partnership team to gain further understanding of and recommendations for KM in a CBO.

**Table 1:** Measures

Measure	Definition	Use in KM research and practice	Recommendation to the CBO based on results of network analysis
Network level measures			
Density	The number of connections in the network as a proportion of all possible ties in the network.	Determine how or if potential information channels are being used in the network.	Efforts to improve information sharing among the network may be needed.
Reciprocity	Assesses the proportion of mutual ties within the network.	Determine if information has been miscommunicated or communicated ineffectively.	Information is being miscommunicated in the network. Efforts to ensure information is provided *and* received effectively are needed.
Core-periphery structure	Network structure that consists of a group of nodes who are densely connected to one another (the core) and a separate group of nodes that are loosely connected to the core (periphery).	Extends the notion of centrality to groups. Characteristics of the core and the periphery can be examined and compared.	Efforts to improve information sharing between members of the core and the periphery as well as between members of the periphery are neeeded.
Individual level measures			
Degree	The number of links to (in-degree) and from (out-degree) an individual.	Identify individuals who are prominent (in-degree) or influential (out-degree).	Managers and coordinators are influential opinion leaders in the network and should be used to disseminate information.
Closeness	The average distance an individual is to (in-closeness) or from (out-closeness) all other individuals in the network.	Identify individuals that can efficiently disseminate information (out-closeness) or be quickly reached (in-closeness).	CBO staff in managerial or coordinator position and peer support volunteers should be used to disseminate information efficiently.
Betweenness	The frequency with which an individual lies on the shortest path connecting all individuals in the network.	Identify potential gatekeepers of information and partnerships.	CBO managers or coordinators are gatekeepers of information and should be encouraged to disseminate information.

### Participants

In total, 81 participants completed the network survey. Approximately 70 % of the sample were staff (*M*_age_ = 42.25 ± 12.26 years), and 30 % (*n* = 34) were volunteers (*M*_age_ = 46.30 ± 11.93 years). Staff were predominantly female (78 %) and 24 % had an SCI. The majority of participants had completed at least one postsecondary degree (93 %). Volunteers were predominantly peer support volunteers who had an SCI (82 %) and who mentored clients with an SCI. The remaining volunteers were family support volunteers who mentored families of someone with an SCI. The majority of the volunteers were male (64 %) and had completed least one postsecondary degree (66 %). On average, volunteers had been affiliated with the volunteer program for 7.60 ± 5.54 years and had mentored 7.47 ± 9.77 peers whereas staff had worked for the CBO for 4.56 ± 5.03 years.

### Network Analysis

In total, the 81 participants named 238 people or organizations in the CBO’s physical activity information sharing network, and 409 connections or ties were reported. If an individual was named but did not respond to the network survey, they were included in the analysis. To further illustrate the value of network analysis for understanding KM, network and individual level results and interpretation are presented below.

#### Density

The CBO’s information sharing network had a density of 3 %, indicating that 3 % of the possible ties within the network were being used [26, 27]. Although we lack a comparison group, these data suggest that the network density is low. Information sharing among the network is likely constrained, and efforts to improve information sharing among the network may be needed [27].

#### Reciprocity

Reciprocity in the network was determined by assessing the percentage of mutual ties (i.e. participants both indicated that they shared information with each other) in the network. The reciprocity within this network was 10 %, indicating that only 10 % of the ties were confirmed or mutual. The low reciprocity in the CBO’s network indicates that physical activity information may be miscommunicated or communicated ineffectively within the network. For example, an individual may believe that he or she has provided information to a number of individuals, but few people indicate that they actually received this information. Greater efforts are likely needed to ensure that information is provided effectively.

#### Core-Periphery Structure

The network structure was shown to be a core-periphery structure [27]. The density of ties among the core actors was 10 %; the density of ties sharing information from the core to the periphery was 3 %; the density sharing information from the periphery to the core was 0.1 %; the density of ties sharing information among periphery actors was 0.1 % (test fitness = 0.28). This pattern of densities indicates that the information sharing among members of the core is more efficient than information sharing between the core and periphery as well as within the periphery. Notably, individuals in the core were primarily staff (97 %) whereas the periphery was primarily comprised of individuals outside the CBO (40 %), volunteers (27 %) and staff (25 %). Individuals in the core were significantly more likely to report being university educated (*p* < 0.05). Further efforts may be needed to improve information sharing between members of the core and the periphery as well as between members of the periphery.

#### Degree Centrality

The range of out-degree scores was wide. However, individuals reported on average sharing information with two individuals (i.e., had an out-degree score of 2; see Table 2). Nine individuals had an out-degree score greater than or equal to 10 and were likely influential in the network. All of these individuals were CBO staff, and 77 % of these individuals worked in client services. These individuals either worked as managers or coordinators with SCI Ontario.

**Table 2:** Centrality scores

	Mean score (standard deviation)	Minimum score	Maximum score
Out-degree	1.71 (8.51)	0	97.00
In-degree	1.71 (1.51)	0	8.00
In-closeness	0.46 (.02)	0.42	0.48
Out-closeness	0.60 (.53)	0.42	2.29
Betweenness	0.09 (.47)	0	4.39

In-degree scores of actors within the CBO’s physical activity information sharing network were less variable than out-degree scores; however, individuals were named by others in the network an average of two times (Table 2). Seven individuals had an in-degree score greater than or equal to 10. These individuals are likely opinion leaders in the network. One of these individuals was a volunteer while all others named were SCI Ontario staff who worked in service provision.

#### Closeness

Large variability in out-closeness scores was observed (Table 2). A group of 24 individuals had out-closeness scores greater than 2 indicating that these individuals could disseminate information efficiently in the network. The majority of these individuals were SCI Ontario staff (83 %) in managerial or coordinator position, and 12 % were peer support volunteers. Little variability in in-closeness scores was observed. Individuals with higher in-closeness scores (i.e. can be quickly reached in the network) were peer support volunteers, researchers and health care professionals.

#### Betweenness

While the majority of individuals within the SCI Ontario network had a betweenness score of 0 indicating that they did not lie on the shortest path connecting all nodes in the network, five individuals within the network had a betweenness score of greater than 1. These individuals had the highest betweenness scores in the network and therefore, are likely potential gatekeepers of information and partnerships in the network and were all SCI Ontario managers or coordinators. Two of these individuals worked specifically in peer support.

## Discussion

The present paper aimed to demonstrate the feasibility and value of network analysis to examine KM in a CBO by highlighting solutions to challenges that could affect the feasibility of adopting a network analysis approach and presenting an example of a network analysis conducted within a CBO. Regarding feasibility, each of the benchmarks set by the CBO were met. Our example analysis provides a description of an organization undergoing KM including identifying prominent or influential individuals and groups as well as highlights areas for improvement in future KM initiatives. Overall, findings demonstrate how network analysis can be feasibly used to provide a rich description of a CBO engaging in KM activities.

Feasibility results highlight the partnership team’s ability to overcome challenges inherent to conducting a network analysis within a CBO. The strong partnership ensured that practically relevant and culturally sensitive network questions were asked; trust and ownership within the CBO were fostered; and face validity of the network instrument was achieved. Nevertheless, differences in participation among the CBO staff and volunteers were observed, and in subsequent sub-analyses, volunteers were removed from the network [23]. The staff’s greater level of participation compared to volunteers may be due to situational and systematic factors. While no remuneration was provided to participants for completing the network survey, it is possible that staff, as paid employees, felt more invested in completing the survey than volunteers. Staff may also pay greater attention and have greater access to organization e-mails and requests than volunteers. Based on these findings, we strongly encourage researchers to consider a partnership approach in which expectations for research participation among CBO members are clearly established and the research objectives are realistic. We also recommend that they consider multiple methods of survey dissemination, such as telephone and online surveys as well as remuneration for volunteers.

The example analysis provides an illustration of how network analysis can provide an understanding of the process of KM within a CBO. The example also highlights how network analysis can be used to enhance future KM interventions within a CBO by fostering an efficient and effective flow of information. For example, the network analysis indicated that density and reciprocity within the CBO network examined were low. While this result may be a function of missing data, results may also provide an indication that future KM efforts aiming to ensure that information is disseminated and received are likely needed. At the individual level, the network analysis identified individuals who are prominent sources of information, have greater opportunities to collaborate and share resources within the network and are gatekeepers of information and partnerships [26, 27].

The results of the network analysis were useful for guiding and informing strategic planning and decision making within the organization. Results provided the organization with an understanding of the existing communication structure within their organization, evidence demonstrating their capacity to reach people with SCI and highlighted areas for expansion and learning opportunities. Prior to the analysis, the CBO was not aware of the low density and reciprocity within the network. Following the analysis, the CBO used the results to improve communication by identifying key opinion leaders and enhancing communication among peripheral members of the network. For example, results of the network analysis led to the development of e-learning activities for staff and volunteers as well as a training for CBO volunteers to aid in their communication about the physical activity guidelines [19]. Overall, the results of the network analysis helped the CBO to meet their strategic priority of becoming a reliable voice for people with SCI and the researchers to understand how KM strategies should be designed to optimize information dissemination and uptake.

Despite our valuable findings, a few limitations of our methodology must be acknowledged. First, we did not have a comparison group; therefore, we could not empirically test that our methodology is superior to other approaches such as conducting the survey by telephone or in person. Second, the network analysis was conducted at one time point. Accordingly, we cannot comment on how the results of the network analysis or the feasibility our methodology evolve over time as participants become more familiar with network surveys. Finally, we could only assess the face validity of our network instrument and not the test-retest reliability of the instrument.

In conclusion, the description of our methodology and results of our analysis demonstrate how network analysis can be used to understand and facilitate the process of KM within CBOs. The community-university multidisciplinary partnership approach used to design, evaluate and implement the network analysis is a foremost strength of this work. By working in partnership, the team was able to address each of the ethical and practical concerns outlined by the CBO and conduct the network analysis. More broadly, this methodology has potential to provide valuable information about CBO network structure and key individuals that can be used to facilitate and understand the process KM in CBO.
